# Supplementary material for: Development of Blue Phosphorescent Pt(II) Materials Using Dibenzofuranyl Imidazole Ligands and Their Application in Organic Light-Emitting Diodes
Source: Materials (Basel). 2023 Jun 2;16(11):4159. doi: 10.3390/ma16114159 (PMC10254305; doi:10.3390/ma16114159)
Supplement: Supplementary file 1 [file materials-16-04159-s001.zip › materials-2395513-supplementary.pdf]

Development of Blue Phosphorescent Pt(II) Materials Using Dibenzofuranyl Imidazole Ligands and Their Application in Organic Light-Emitting Diodes

Hakjo Kim [1], Dain Cho [1], Haein Kim [1], Seung Chan Kim [2], Jun Yeob Lee [2,*] and Youngjin Kang [1,*]

[1] Division of Science Education, Kangwon National University, Chuncheon 24341, Republic of Korea; tank528@naver.com (H.K.); dain0041@naver.com (D.C.); godls0126@naver.com (H.K.)

[2] School of Chemical Engineering, Sungkyunkwan University, 2066 Seobu-ro, Jangan-gu, Suwon 16419, Republic of Korea; ksc9650@naver.com

* Correspondence: leej17@skku.edu (J.Y.L.); kangy@kangwon.ac.kr (Y.K.)

Contents

- Experimental details.
- **Figure S1**. ^{1}H NMR of **1** in CD_2Cl_2.
- **Figure S2.** ^{13}C NMR of **1** in CD_2Cl_2.
- **Figure S3**. ^{1}H NMR of **2**in CD_2Cl_2.
- **Figure S4.** ^{13}C NMR of **2** in CD_2Cl_2
- **Figure S5.** X-ray structure of **2** using Diamond program.
- **Figure S6.** Decay curve of **1.**
- **Figure S7.** Decay curve of **2** at different wavelngth (black: 482nm, red:435nm).
- **Figure S8.** Oxidation of **1** and **2** (inset: Fc/Fc+ oxidation).
- **Figure S9.** Expanded ^{3}MLCT absorption band of **1** and **2** at 460 nm ~ 480nm.
- **Table S1.** Crystal Data and Structure Refinement for **2**.
- **Table S2.** Atomic coordinates (x 10^4) and equivalent isotropic displacement parameters ($Å^2$x 10^3) for **2**. U(eq) is defined as one third of the trace of the orthogonalized U^{ij} tensor.
- **Table S3**. Bond lengths [Å] and angles [°] for **2**.
- **Table S4**. Anisotropic displacement parameters ($Å^2$x 10^3) for **2**. The anisotropic displacement factor exponent takes the form: -2 ^{2}[h^2 $a^{*2}U^{11}$ + ... + 2 h k a* b* U^{12}]
- **Table S5.** Hydrogen coordinates (x 10^4) and isotropic displacement parameters

($Å^2$x 10 ^{3}) for **2**.

- **Table S6**. Hydrogen bonds for **2** [Å and °].
- **Table S7**. TD-DFT calculation results of **1** and **2**.
- **Table S8.** PHOLEDs data of **1**-based devices at different doing levels.

Experimental Details

2.1. General Consideration

All experiments were performed under N_2 atmosphere using standard Schlenk technique. Solvents used were dried over suitable drying agents prior to use. All starting materials were commercially purchased and used without further purification. The spectra of NMR were recorded on a 400 or 600 MHz JEOL or Bruker FT-NMR spectrometer. The absorption and emission spectra were obtained using UV/Vis spectrometer Shimadzu UV-1601 and Perkin Elmer Luminescence spectrometer LS 50B, respectively. The transient PL decay curve and absolute PL quantum yield were measured using Quantaurus-Tau system (Hamamatsu, C11367-31) and Quantaurus-QY system (Hamamatsu, C11347-11) under N_2 atmosphere, respectively. All solvents for measuring photophysical properties were degassed with nitrogen before use.

2.2. X-ray crystallographic analysis

X-ray diffraction data for complex **2** were collected at 173(2) K on a Bruker SMART APEX II ULTRA diffractometer equipped with a graphite monochromated Mo Kα (λ= 0.71073 Å) radiation generated by a rotating anode and a CCD detector. The cell parameters for the compound were obtained from a least-squares refinement of the spots (from 36 collected frames). Data collection, data reduction, and semi-empirical absorption correction (SADABS) were carried out using the software package of APEX2. All of the calculations for the structure determination were carried out using the APEX2 package with the SHELXS-2014 and SHELXL-2014 programs. The non-hydrogen atoms of both compounds were refined anisotropically. All hydrogen atoms were placed in calculated positions and refined isotropically in a riding manner along with their respective parent atoms. The figure was prepared using Diamond program. A summary of the refinement details and resulting factors for the crystal structures of **2** are given in Table S1. CCDC-2259034 contains the supplementary crystallographic data for this paper. These data can be obtained free of charge from The Cambridge Crystallographic Data Centre via www.ccdc.cam.ac.uk/data_request/cif.

2.3. Device Fabrication and Measurement

The compound **1** phosphor was evaluated by doping in the mixed host, 3,3′-di(9H-carbazol-9-yl)-1,1′-biphenyl(mCBP)/ 9-(3'-Carbazol-9-yl-5-cyano-biphenyl-3-yl) -9H-carbazole-3-carbonitrile (CNmCBP CN) at 3 and 10% using the device configuration of indium tin oxide (50 nm) /poly(3,4-ethylenedioxythiophene) (PEDOT, 40 nm)/4,4′-cyclohexylidenebis[*N*,*N*-bis(4-methylphenyl)benzene amine] (TAPC, 10 nm) / 1,3-bis(*N*-carbazolyl)benzene (mCP, 10 nm) / mCBP:CNmCBPCN dopant (25 nm) / diphenyl[4-(triphenylsilyl)phenyl]phosphine oxide (TSPO1, 5 nm) / 1,3,5–tris (1-phenyl -1*H*-benzo[*d*]imidazol-2-yl)benzene (TPBi, 20 nm) / LiF (1.5 nm) / Al (200 nm).

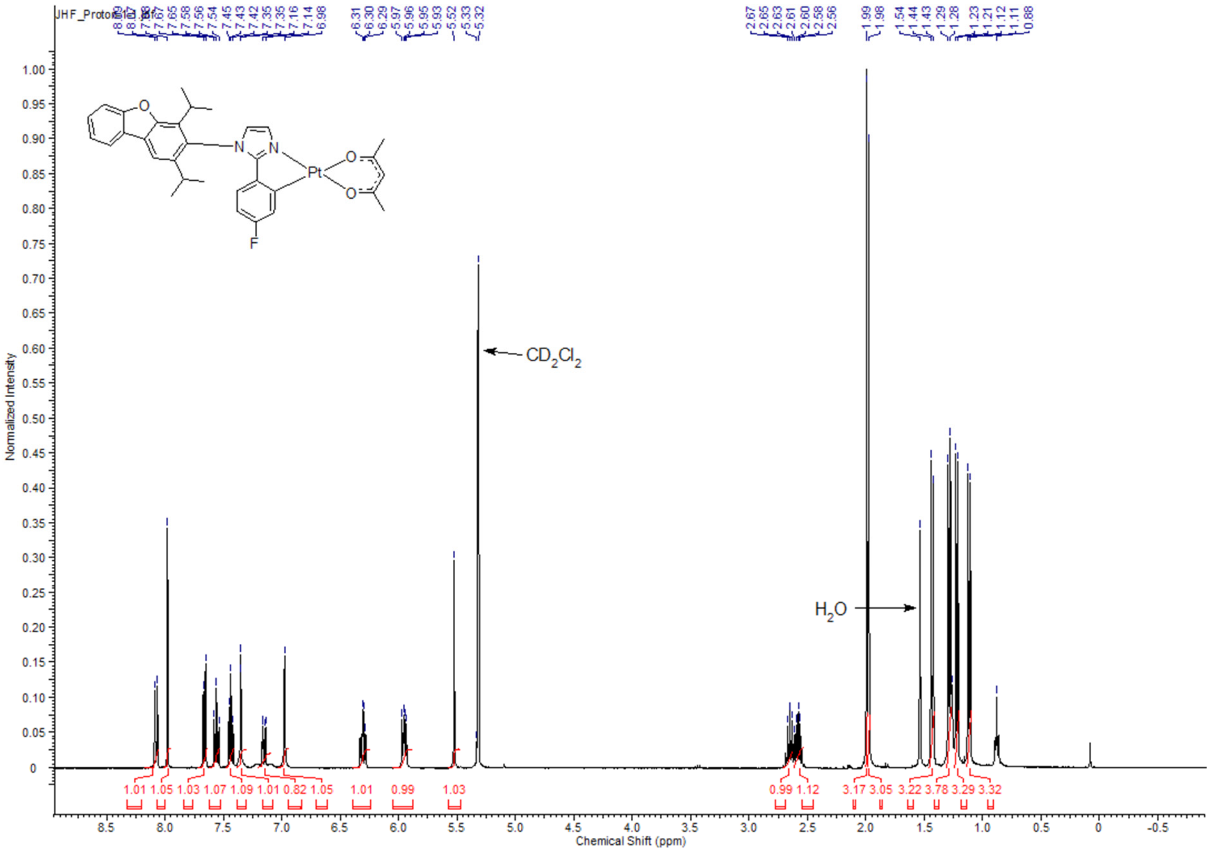

Figure S1. ^{1}H NMR of **1** in CD_2Cl_2.

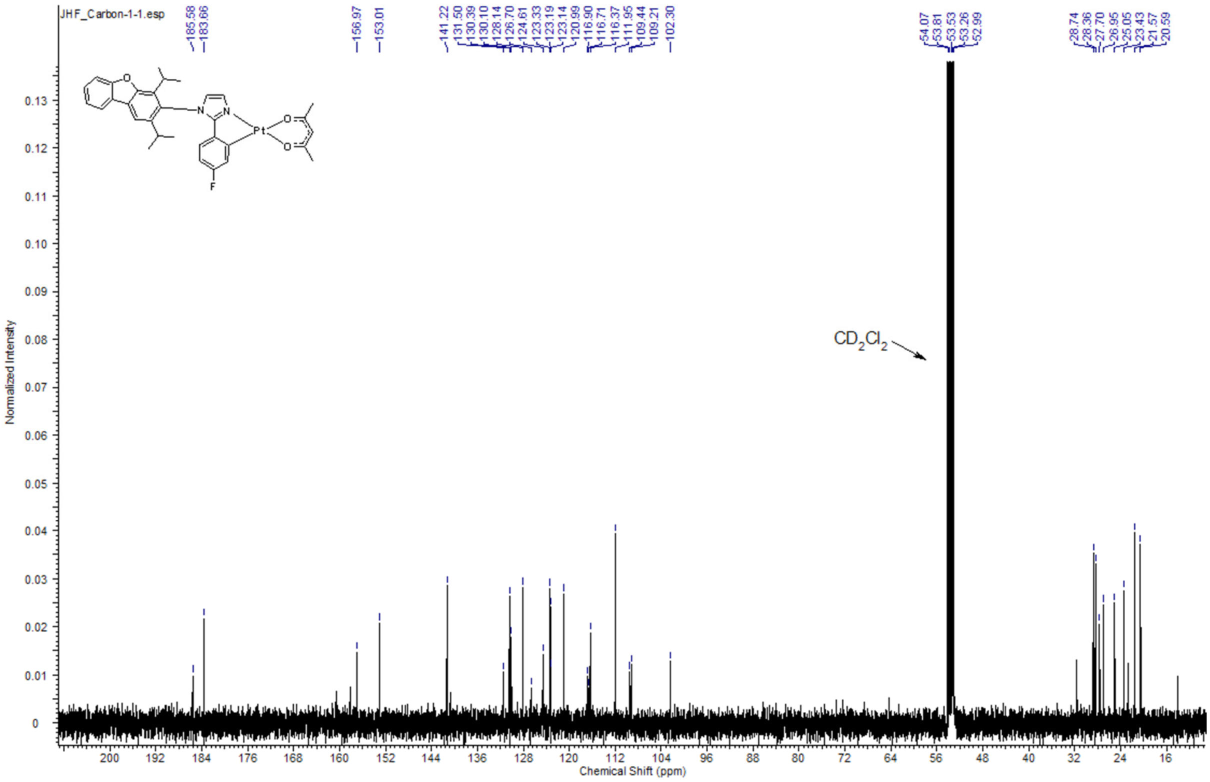

Figure S2. ^{13}C NMR of **1** in CD_2Cl_2**.**

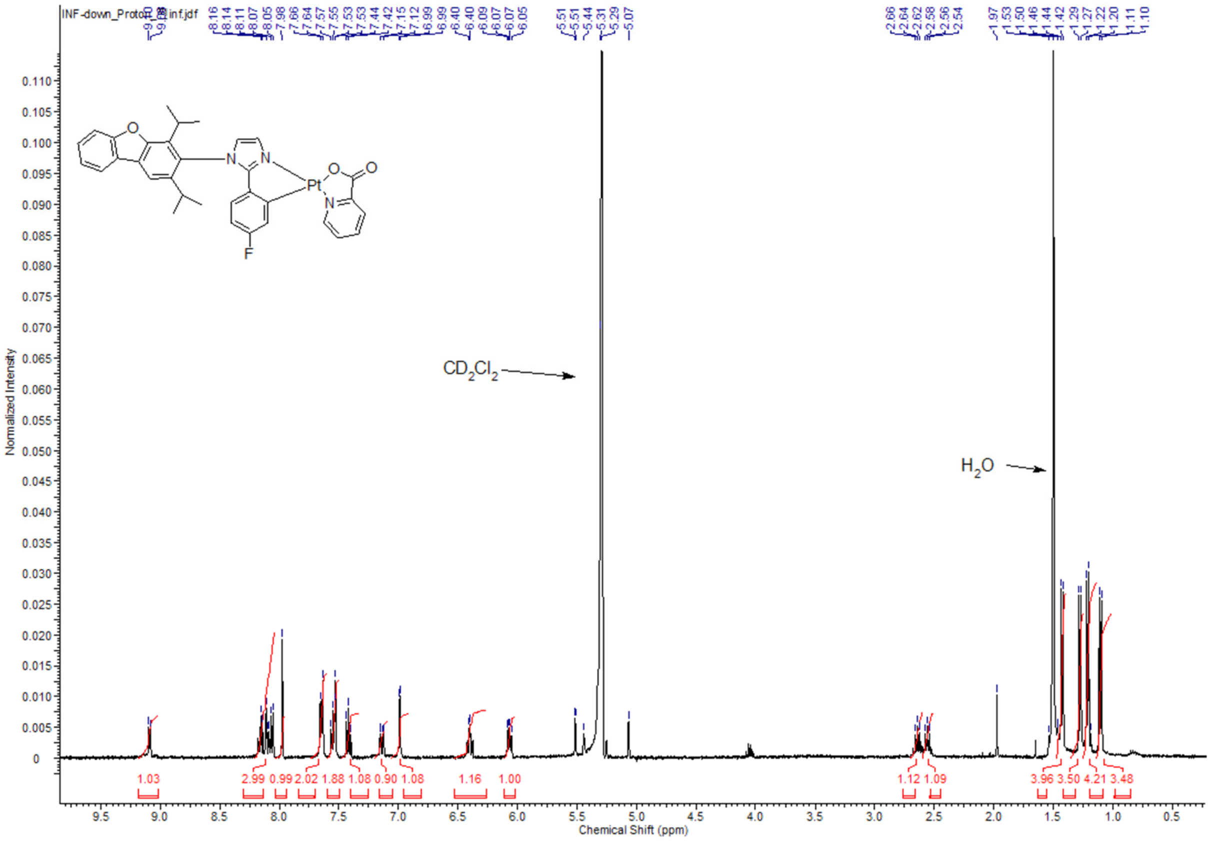

Figure S3. ^{1}H NMR of **2**in CD_2Cl_2.

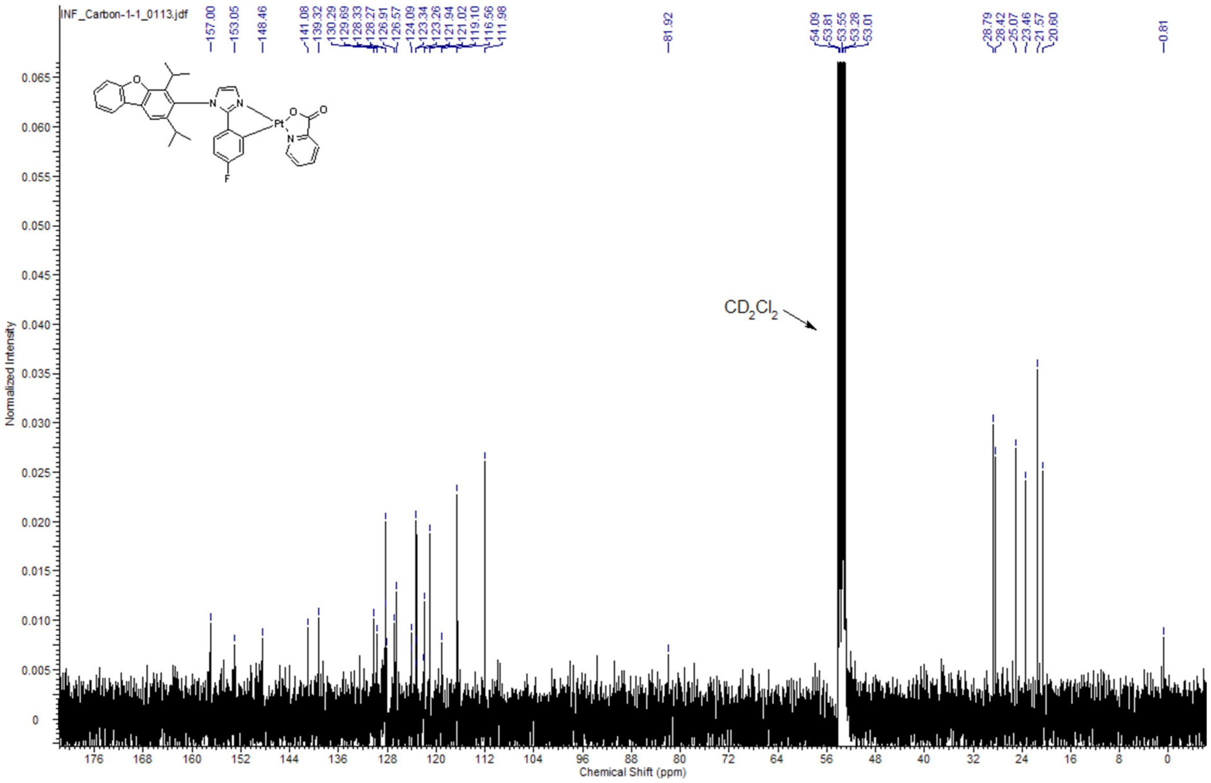

Figure S4. ^{13}C NMR of **3** in CD_2Cl_2

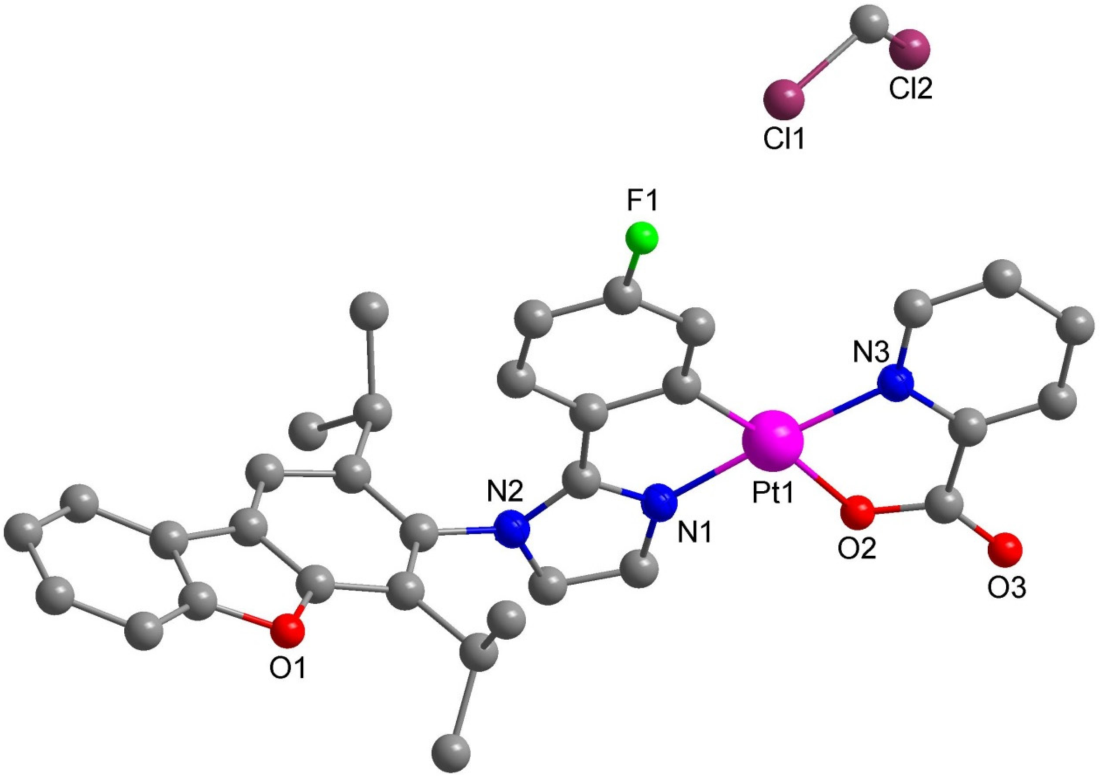

Figure S5. X-ray structure of **2** using Diamond program.

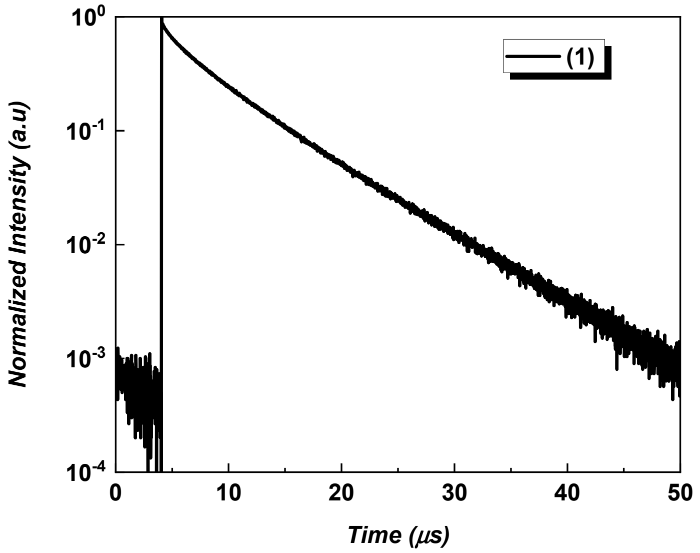

Figure S6. Decay curve of **1.**

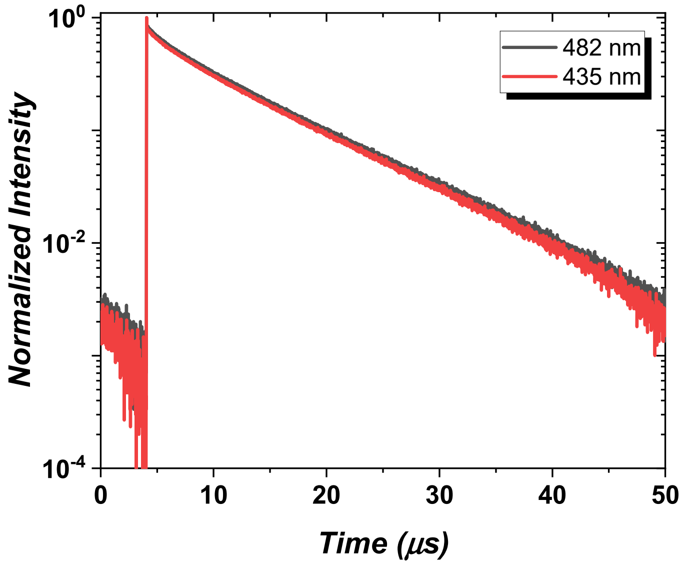

Figure S7. Decay curve of **2** at different wavelngth (black: 482nm, red:435nm).

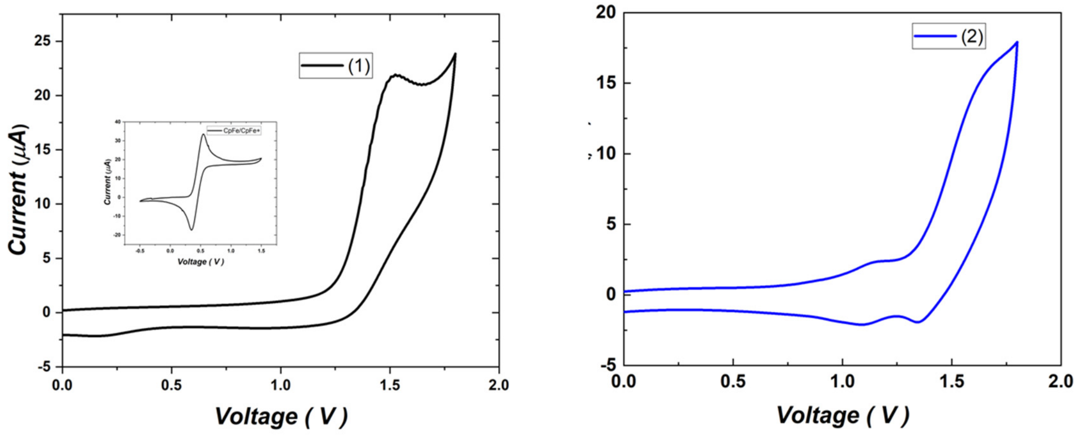

Figure S8. Oxidation of **1** (left) and **2** (right)(inset: Fc/Fc+ oxidation).

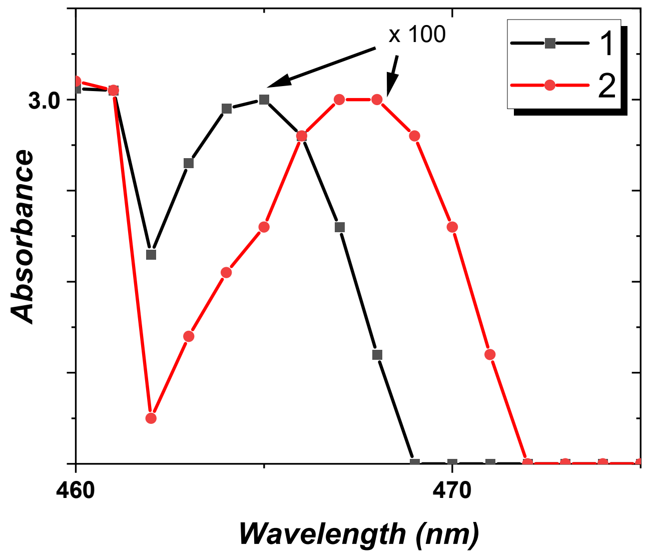

Figure S9. Expanded ^{3}MLCT absorption band of **1** and **2** at 460 nm ~ 480nm.

Table S1. Crystal Data and Structure Refinement for 2.

Identification code	**2**	
Empirical formula	C34 H30 Cl2 F N3 O3 Pt	
	$[Pt(C_{27}H_{24}N_2OF)(C_6H_4NO_2)]\cdot(CH_2Cl_2)$	
Formula weight	813.60	
Temperature	173(2) K	
Wavelength	0.71073 Å	
Crystal system	Monoclinic	
Space group	$P2_1/n$	
Unit cell dimensions	a = 11.3734(3) Å	= 90°.
	b = 13.4201(4) Å	= 101.2050(10)°.
	c = 20.8322(6) Å	= 90°.
Volume	3119.05(15) $Å^3$	
Z	4	
Density (calculated)	1.733 Mg/m^3	
Absorption coefficient	4.716 mm^{-1}	
F(000)	1600	
Crystal size	0.298 x 0.222 x 0.093 mm^3	
Theta range for data collection	1.815 to 28.324°.	
Index ranges	-15<=h<=15, -17<=k<=17, -27<=l<=27	
Reflections collected	82770	
Independent reflections	7757 [R(int) = 0.0293]	
Completeness to theta = 25.242°	100.0 %	
Absorption correction	Semi-empirical from equivalents	
Max. and min. transmission	0.7457 and 0.5114	
Refinement method	Full-matrix least-squares on F^2	
Data / restraints / parameters	7757 / 0 / 397	
Goodness-of-fit on F^2	1.105	
Final R indices [I>2sigma(I)]	R1 = 0.0228, wR2 = 0.0547	
R indices (all data)	R1 = 0.0262, wR2 = 0.0561	
Extinction coefficient	n/a	
Largest diff. peak and hole	1.090 and -0.469 e.$Å^{-3}$	

Table S2. Atomic coordinates (x 10^4) and equivalent isotropic displacement parameters ($Å^2$x 10^3) for **2**. U(eq) is defined as one third of the trace of the orthogonalized U^{ij} tensor.

	x	y	z	U(eq)
Pt(1)	4718(1)	5950(1)	7676(1)	27(1)
F(1)	5305(2)	3582(2)	5649(1)	64(1)
N(1)	5936(2)	6996(2)	7642(1)	29(1)
N(2)	7339(2)	7684(2)	7212(1)	32(1)
N(3)	3413(2)	4962(2)	7777(1)	29(1)
O(1)	11141(2)	6752(2)	6671(1)	41(1)
O(2)	4272(2)	6497(2)	8529(1)	40(1)
O(3)	3411(3)	5982(2)	9341(1)	67(1)
C(1)	5348(2)	5476(2)	6892(1)	28(1)
C(2)	5078(3)	4622(2)	6519(2)	36(1)
C(3)	5614(3)	4438(2)	5990(2)	40(1)
C(4)	6434(3)	5047(3)	5799(2)	44(1)
C(5)	6751(3)	5899(2)	6165(2)	42(1)
C(6)	6236(3)	6106(2)	6707(1)	32(1)
C(7)	6533(2)	6937(2)	7154(1)	30(1)
C(8)	6367(3)	7800(2)	8023(1)	35(1)
C(9)	7242(3)	8228(2)	7761(2)	34(1)
C(10)	8265(3)	7792(2)	6830(2)	35(1)
C(11)	8032(3)	8422(2)	6273(2)	40(1)
C(12)	8891(3)	8461(2)	5880(2)	41(1)
C(13)	9900(3)	7896(2)	6044(1)	36(1)
C(14)	10100(3)	7299(2)	6596(2)	36(1)
C(15)	9288(3)	7225(2)	7028(2)	36(1)
C(16)	10947(3)	7699(2)	5737(2)	38(1)
C(17)	11317(3)	8041(3)	5170(2)	48(1)
C(18)	12377(3)	7667(3)	5036(2)	50(1)
C(19)	13046(3)	6979(3)	5453(2)	49(1)
C(20)	12678(3)	6626(3)	6010(2)	45(1)
C(21)	11627(3)	7006(2)	6129(2)	40(1)
C(22)	6894(4)	9012(3)	6087(2)	52(1)
C(23)	6125(5)	8628(5)	5450(3)	97(2)
C(24)	7154(5)	10116(3)	6040(3)	91(2)
C(25)	9496(3)	6563(2)	7627(2)	44(1)

C(26)	10646(4)	6821(3)	8106(2)	58(1)
C(27)	9477(4)	5469(3)	7435(2)	60(1)
C(28)	3626(3)	5892(2)	8796(2)	40(1)
C(29)	3074(2)	5042(2)	8363(1)	32(1)
C(30)	2219(3)	4431(3)	8541(2)	40(1)
C(31)	1675(3)	3710(2)	8106(2)	42(1)
C(32)	1992(3)	3643(2)	7505(2)	42(1)
C(33)	2849(3)	4291(2)	7354(2)	39(1)
C(34)	866(4)	3565(3)	5455(2)	67(1)
Cl(1)	2400(1)	3734(1)	5522(1)	90(1)
Cl(2)	222(1)	4472(1)	5892(1)	83(1)

Table S3. Bond lengths [Å] and angles [°] for **2**.

Pt(1)-N(1)	1.983(2)
Pt(1)-C(1)	2.009(3)
Pt(1)-N(3)	2.032(2)
Pt(1)-O(2)	2.075(2)
F(1)-C(3)	1.360(4)
N(1)-C(7)	1.331(3)
N(1)-C(8)	1.372(4)
N(2)-C(7)	1.347(3)
N(2)-C(9)	1.379(4)
N(2)-C(10)	1.446(3)
N(3)-C(33)	1.334(4)
N(3)-C(29)	1.354(3)
O(1)-C(14)	1.376(4)
O(1)-C(21)	1.393(4)
O(2)-C(28)	1.290(4)
O(3)-C(28)	1.213(4)
C(1)-C(2)	1.385(4)
C(1)-C(6)	1.427(4)
C(2)-C(3)	1.381(4)
C(3)-C(4)	1.357(4)
C(4)-C(5)	1.384(4)
C(5)-C(6)	1.397(4)
C(6)-C(7)	1.450(4)
C(8)-C(9)	1.352(4)
C(10)-C(15)	1.384(4)
C(10)-C(11)	1.420(4)
C(11)-C(12)	1.392(4)
C(11)-C(22)	1.503(5)
C(12)-C(13)	1.362(4)
C(13)-C(14)	1.383(4)
C(13)-C(16)	1.481(4)
C(14)-C(15)	1.413(4)
C(15)-C(25)	1.512(4)
C(16)-C(21)	1.373(5)
C(16)-C(17)	1.407(5)
C(17)-C(18)	1.383(5)

C(18)-C(19)	1.389(5)
C(19)-C(20)	1.391(5)
C(20)-C(21)	1.365(4)
C(22)-C(24)	1.519(6)
C(22)-C(23)	1.531(6)
C(25)-C(27)	1.521(5)
C(25)-C(26)	1.523(5)
C(28)-C(29)	1.514(4)
C(29)-C(30)	1.377(4)
C(30)-C(31)	1.386(5)
C(31)-C(32)	1.373(5)
C(32)-C(33)	1.386(4)
C(34)-Cl(1)	1.737(5)
C(34)-Cl(2)	1.762(5)

N(1)-Pt(1)-C(1)	80.44(10)
N(1)-Pt(1)-N(3)	174.37(9)
C(1)-Pt(1)-N(3)	105.10(10)
N(1)-Pt(1)-O(2)	93.94(9)
C(1)-Pt(1)-O(2)	173.25(10)
N(3)-Pt(1)-O(2)	80.60(9)
C(7)-N(1)-C(8)	108.1(2)
C(7)-N(1)-Pt(1)	116.66(18)
C(8)-N(1)-Pt(1)	135.22(19)
C(7)-N(2)-C(9)	107.8(2)
C(7)-N(2)-C(10)	125.5(2)
C(9)-N(2)-C(10)	126.0(2)
C(33)-N(3)-C(29)	117.7(2)
C(33)-N(3)-Pt(1)	129.8(2)
C(29)-N(3)-Pt(1)	112.55(19)
C(14)-O(1)-C(21)	104.3(2)
C(28)-O(2)-Pt(1)	113.68(19)
C(2)-C(1)-C(6)	115.4(2)
C(2)-C(1)-Pt(1)	130.0(2)
C(6)-C(1)-Pt(1)	114.53(19)
C(3)-C(2)-C(1)	120.8(3)
C(4)-C(3)-F(1)	118.5(3)
C(4)-C(3)-C(2)	124.1(3)

F(1)-C(3)-C(2)	117.3(3)
C(3)-C(4)-C(5)	117.2(3)
C(4)-C(5)-C(6)	120.3(3)
C(5)-C(6)-C(1)	122.1(3)
C(5)-C(6)-C(7)	126.1(3)
C(1)-C(6)-C(7)	111.8(2)
N(1)-C(7)-N(2)	109.0(2)
N(1)-C(7)-C(6)	116.5(2)
N(2)-C(7)-C(6)	134.4(2)
C(9)-C(8)-N(1)	108.0(2)
C(8)-C(9)-N(2)	107.1(2)
C(15)-C(10)-C(11)	125.7(3)
C(15)-C(10)-N(2)	116.2(3)
C(11)-C(10)-N(2)	118.0(3)
C(12)-C(11)-C(10)	117.6(3)
C(12)-C(11)-C(22)	119.8(3)
C(10)-C(11)-C(22)	122.6(3)
C(13)-C(12)-C(11)	118.9(3)
C(12)-C(13)-C(14)	121.8(3)
C(12)-C(13)-C(16)	134.3(3)
C(14)-C(13)-C(16)	103.9(3)
O(1)-C(14)-C(13)	113.7(3)
O(1)-C(14)-C(15)	123.0(3)
C(13)-C(14)-C(15)	123.2(3)
C(10)-C(15)-C(14)	112.8(3)
C(10)-C(15)-C(25)	123.8(3)
C(14)-C(15)-C(25)	123.4(3)
C(21)-C(16)-C(17)	119.6(3)
C(21)-C(16)-C(13)	105.8(3)
C(17)-C(16)-C(13)	134.6(3)
C(18)-C(17)-C(16)	117.7(3)
C(17)-C(18)-C(19)	120.6(3)
C(18)-C(19)-C(20)	122.1(3)
C(21)-C(20)-C(19)	116.1(3)
C(20)-C(21)-C(16)	123.9(3)
C(20)-C(21)-O(1)	123.7(3)
C(16)-C(21)-O(1)	112.4(3)
C(11)-C(22)-C(24)	111.2(3)

C(11)-C(22)-C(23)	111.3(4)
C(24)-C(22)-C(23)	111.0(4)
C(15)-C(25)-C(27)	111.0(3)
C(15)-C(25)-C(26)	112.6(3)
C(27)-C(25)-C(26)	111.0(3)
O(3)-C(28)-O(2)	125.2(3)
O(3)-C(28)-C(29)	119.8(3)
O(2)-C(28)-C(29)	114.9(2)
N(3)-C(29)-C(30)	122.4(3)
N(3)-C(29)-C(28)	116.0(2)
C(30)-C(29)-C(28)	121.4(3)
C(29)-C(30)-C(31)	119.3(3)
C(32)-C(31)-C(30)	118.6(3)
C(31)-C(32)-C(33)	119.1(3)
N(3)-C(33)-C(32)	122.9(3)
Cl(1)-C(34)-Cl(2)	112.3(2)

Symmetry transformations used to generate equivalent atoms:

Table S4. Anisotropic displacement parameters (Å^{2}x 10^3) for **2**. The anisotropic displacement factor exponent takes the form: -2 ^{2}[h^2 a*^{2}U^{11} + ... + 2 h k a* b* U^{12}]

	U^{11}	U^{22}	U^{33}	U^{23}	U^{13}	U^{12}
Pt(1)	25(1)	28(1)	28(1)	-2(1)	9(1)	0(1)
F(1)	75(2)	54(1)	73(1)	-36(1)	45(1)	-25(1)
N(1)	28(1)	28(1)	33(1)	-4(1)	10(1)	-1(1)
N(2)	31(1)	29(1)	38(1)	-2(1)	12(1)	-2(1)
N(3)	26(1)	30(1)	32(1)	1(1)	9(1)	3(1)
O(1)	39(1)	41(1)	43(1)	4(1)	11(1)	4(1)
O(2)	42(1)	44(1)	38(1)	-12(1)	18(1)	-8(1)
O(3)	77(2)	93(2)	39(1)	-19(1)	29(1)	-27(2)
C(1)	27(1)	27(1)	31(1)	0(1)	9(1)	2(1)
C(2)	34(2)	34(1)	42(2)	-5(1)	17(1)	-2(1)
C(3)	41(2)	37(2)	44(2)	-13(1)	16(1)	-3(1)
C(4)	52(2)	47(2)	40(2)	-12(1)	24(1)	-9(2)
C(5)	46(2)	47(2)	40(2)	-8(1)	21(1)	-11(1)
C(6)	35(2)	32(1)	31(1)	-2(1)	10(1)	-2(1)
C(7)	29(1)	30(1)	32(1)	0(1)	10(1)	-1(1)
C(8)	34(1)	34(1)	38(1)	-6(1)	11(1)	1(1)
C(9)	32(1)	28(1)	41(2)	-7(1)	8(1)	0(1)
C(10)	34(1)	31(1)	41(2)	-4(1)	15(1)	-7(1)
C(11)	44(2)	37(2)	42(2)	-1(1)	13(1)	-7(1)
C(12)	42(2)	41(2)	41(2)	8(1)	8(1)	-3(1)
C(13)	36(2)	33(1)	36(1)	1(1)	3(1)	-10(1)
C(14)	35(2)	31(1)	40(2)	-3(1)	2(1)	1(1)
C(15)	37(2)	29(1)	44(2)	-3(1)	12(1)	-6(1)
C(16)	33(2)	41(2)	44(2)	-12(1)	12(1)	-13(1)
C(17)	42(2)	50(2)	51(2)	-6(2)	9(2)	-8(2)
C(18)	45(2)	58(2)	51(2)	-13(2)	22(2)	-12(2)
C(19)	40(2)	59(2)	54(2)	-16(2)	21(2)	-8(2)
C(20)	42(2)	49(2)	47(2)	-8(2)	13(1)	-3(2)
C(21)	38(2)	45(2)	39(2)	-9(1)	12(1)	-9(1)
C(22)	51(2)	58(2)	51(2)	15(2)	14(2)	6(2)
C(23)	67(3)	102(4)	106(4)	-7(4)	-21(3)	11(3)
C(24)	76(3)	48(2)	146(5)	12(3)	12(3)	12(2)
C(25)	40(2)	38(2)	55(2)	5(1)	16(2)	7(1)

C(26)	70(3)	44(2)	53(2)	2(2)	-3(2)	10(2)
C(27)	59(2)	37(2)	80(3)	8(2)	3(2)	-3(2)
C(28)	38(2)	52(2)	31(1)	-7(1)	11(1)	-1(1)
C(29)	29(1)	37(2)	31(1)	5(1)	7(1)	4(1)
C(30)	37(2)	48(2)	37(2)	15(1)	11(1)	4(1)
C(31)	32(2)	38(2)	56(2)	16(1)	8(1)	-2(1)
C(32)	36(2)	35(2)	57(2)	-5(1)	10(1)	-2(1)
C(33)	34(2)	41(2)	44(2)	-7(1)	13(1)	-3(1)
C(34)	79(3)	61(3)	55(2)	0(2)	-2(2)	-30(2)
Cl(1)	82(1)	124(1)	66(1)	-8(1)	23(1)	-34(1)
Cl(2)	103(1)	81(1)	71(1)	13(1)	29(1)	-4(1)

Table S5. Hydrogen coordinates (x 10^4) and isotropic displacement parameters ($Å^2$x 10^3) for **2**.

	x	y	z	U(eq)
H(2)	4517	4158	6628	43
H(4)	6775	4895	5429	53
H(5)	7322	6346	6048	51
H(8)	6098	8018	8404	42
H(9)	7703	8796	7925	40
H(12)	8775	8875	5503	50
H(17)	10854	8513	4888	57
H(18)	12650	7883	4656	59
H(19)	13779	6743	5355	59
H(20)	13131	6149	6292	54
H(22)	6424	8922	6442	63
H(23A)	5390	9025	5342	145
H(23B)	6576	8684	5095	145
H(23C)	5916	7928	5503	145
H(24A)	6398	10482	5919	137
H(24B)	7591	10356	6464	137
H(24C)	7642	10223	5707	137
H(25)	8815	6675	7858	52
H(26A)	10740	6373	8484	87
H(26B)	11332	6744	7890	87
H(26C)	10606	7512	8253	87
H(27A)	9614	5056	7830	90
H(27B)	8695	5305	7165	90
H(27C)	10109	5341	7186	90
H(30)	2004	4503	8957	48
H(31)	1094	3272	8222	50
H(32)	1630	3160	7196	51
H(33)	3043	4254	6931	47
H(34A)	717	2896	5623	81
H(34B)	474	3593	4988	81

Table S6. Hydrogen bonds for **2** [Å and °].

D-H...A	d(D-H)	d(H...A)	d(D...A)	<(DHA)
C(4)-H(4)...Cl(1)#1	0.95	2.99	3.665(3)	129.6
C(33)-H(33)...Cl(1)	0.95	2.97	3.823(3)	150.7
C(34)-H(34A)...O(2)#2	0.99	2.58	3.512(5)	157.6

Symmetry transformations used to generate equivalent atoms:

#1 -x+1,-y+1,-z+1 #2 -x+1/2,y-1/2,-z+3/2

Table S7. TD-DFT calculation results of **1** and **2**.

	Transition	λ/E (nm/eV)	f	MO	CI	%	MLCT(%)
**1**	$S_0 \rightarrow S_1$	355.02/3.4924	0.0031	HOMO → LUMO	0.6926	95.9	36.6
	$S_0 \rightarrow T_1$	422.52/2.9344		HOMO → LUMO	0.6101	74.4	
**2**	$S_0 \rightarrow S_1$	414.65/2.9901	0.0054	HOMO → LUMO	0.6883	94.8	43.1
	$S_0 \rightarrow T_1$	443.81/2.7937		HOMO → LUMO	0.6478	83.9	

Table S8. PHOLEDs data of **1**-based devices at different doing levels.

**Doping %**	**Voltage(V) at 1000 cd/m²**	**Max EQE(%)**	**At 100 cd/m²**			**At 1000 cd/m²**		
			**CIE (*x*, *y*)**	**EQE (%)**	**CE (cd/A)**	**EQE (%)**	**CE (cd/A)**	**PE (lm/W)**
3%	5.9	8.2	(0.19, 0.32)	6.5	13.6	4.3	8.7	4.6
10%	5.9	10.4	(0.19, 0.34)	8.4	18.0	5.4	11.5	10.4
Reference 10%	5.7	9.7	(0.19, 0.37)	9.5	21.7	7.0	15.8	8.8

Device structure: PEDOT(40nm) /TAPC(10m)/ mCP(10nm)/mCBP:CNmCBPCN;Pt(25nm:x%:Pt)/TSPO1(5nm) /TPBi(20nm) /LiF(1.5nm) /Al(200nm). CE: Current Efficiency; PE:Power efficiency: EQE: External Quantum Efficiency. Reference: Pt(tpim)(O^O).
